# Bioclimatic analysis and spatial distribution of fascioliasis causative agents by assessment of Lymnaeidae snails in northwestern provinces of Iran

**DOI:** 10.1186/s13071-024-06298-2

**Published:** 2024-05-31

**Authors:** Hossein Galavani, Ali Haniloo, Saber Raeghi, Mohammad Amin Ghatee, Mehdi Karamian

**Affiliations:** 1 Department of Medical Parasitology and Mycology, School of Medicine, Zanjan University of Medical Sciences, Zanjan, Iran; 2 Department of Medical Parasitology and Mycology, Urmia University of Medical Sciences, Urmia, Iran; 3 Department of Microbiology, School of Medicine, Yasuj University of Medical Sciences, Yasuj, Iran

**Keywords:** *Fasciola*, Lymnaeid snails, GIS, PCR, Iran

## Abstract

**Background:**

Snails of the Lymnaeidae family are the intermediate hosts of *Fasciola* species, the causative agents of fascioliasis. The purpose of this study was to determine the prevalence of *Fasciola* species in lymnaeid snails and to investigate the association of geoclimatic factors and *Fasciola* species distribution in northwestern provinces of Iran using geographical information system (GIS) data.

**Methods:**

A total of 2000 lymnaeid snails were collected from 33 permanent and seasonal habitats in northwestern Iran during the period from June to November 2021. After identification by standard morphological keys, they were subjected to shedding and crushing methods. Different stages of *Fasciola* obtained from these snails were subjected to the *ITS1* polymerase chain reaction–restriction fragment length polymorphism (PCR–RFLP) method for species identification. The associations of weather temperature, rainfall, humidity, evaporation, air pressure, wind speed, elevation, and land cover with the distribution of *Fasciola* species were investigated. Geographical and statistical analysis was performed using ArcMap and SPSS software, respectively, to determine factors related to *Fasciola* species distribution.

**Results:**

Of the 2000 snails collected, 19 were infected with *Fasciola hepatica* (0.09%), six with *F. gigantica* (0.03%), and 13 with other trematodes. Among geoclimatic and environmental factors, mean humidity, maximum humidity, and wind speed were significantly higher in areas where *F. hepatica* was more common than *F. gigantica*. The altitude of *F. hepatica*-prevalent areas was generally lower than *F. gigantica* areas. No significant relationship was observed between other investigated geoclimatic factors and the distribution of infected snails.

**Conclusions:**

The present study showed the relationship of humidity and wind speed with the distribution of snails infected with *F. hepatica* or *F. gigantica* in the northwestern regions of Iran. In contrast to *F. gigantica*, *F. hepatica* was more prevalent in low-altitude areas. Further research is recommended to elucidate the relationship between geoclimatic factors and the presence of intermediate hosts of the two *Fasciola* species.

**Graphical Abstract:**

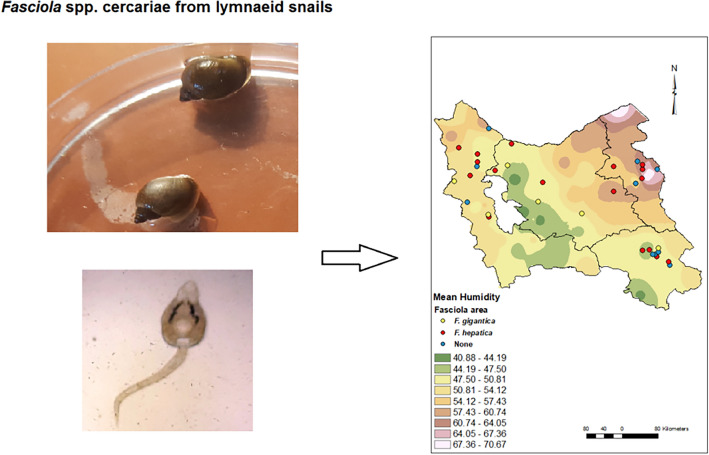

## Background

Fascioliasis is a vector-borne disease transmitted by aquatic and amphibious snails and is caused by either *Fasciola hepatica* or *Fasciola gigantica* of the digenean Platyhelminthes [1]. *Fasciola hepatica* is common in temperate zones such as Asia, Europe, the Americas, and Australia, while *F. gigantica* is limited to the tropical or subtropical regions of Africa, Asia, and the Far East. In contrast, both species overlap in occurrence in subtropical areas [2].

Fascioliasis is a common disease of livestock, and although it is rarer in humans, the estimated prevalence and projected disease burden of human fascioliasis are also significant [3]. According to the World Health Organization (WHO), human fascioliasis has been reported in more than 70 countries, with at least 2.4 million people infected worldwide and several million more at risk (https://www.who.int/publications/i/item/WHO-UCN-NTD-VVE-2021.4). Freshwater snails within the Lymnaeidae family are of great interest due to their participation in the life cycle of various trematodes with biomedical and veterinary importance [1, 4]. About 20 species of lymnaeid snails have been described as the potential intermediate hosts of *Fasciola* spp. Based on the research conducted so far, lymnaeid snails of the genera *Galba* and *Lymnaea*, especially *Galba truncatula* and *Radix euphratica* (syn. *Radix gedrosiana*), are known as the main first intermediate hosts of *Fasciola* spp. in different parts of the world [1]. The epidemiology of fascioliasis is highly related to the ecological characteristics of their snail hosts, while the susceptibility of these intermediate hosts to *Fasciola* species may vary [5]. Research has shown that *F. hepatica* is more common in high and mountainous areas, while *F. gigantica* is more common in lower areas, depending on the distribution of the intermediate snail [6, 7]. Controlling the population of intermediate snail hosts can be considered a suitable strategy to reduce the endemicity of food-borne trematode infections, especially fascioliasis [8–10]. This issue shows the importance of investigating the influence of geographical factors on the distribution of intermediate snail hosts and their infection rate. This study was designed to investigate the influence of environmental and geoclimatic factors on the spread of lymnaeid snails infected with *Fasciola* in the northwestern regions of Iran, which constitute one of the most important centers of agriculture and animal husbandry in Iran, and where animal fascioliasis is also highly prevalent [11].

## Methods

### Study area

**Fig. 1:**
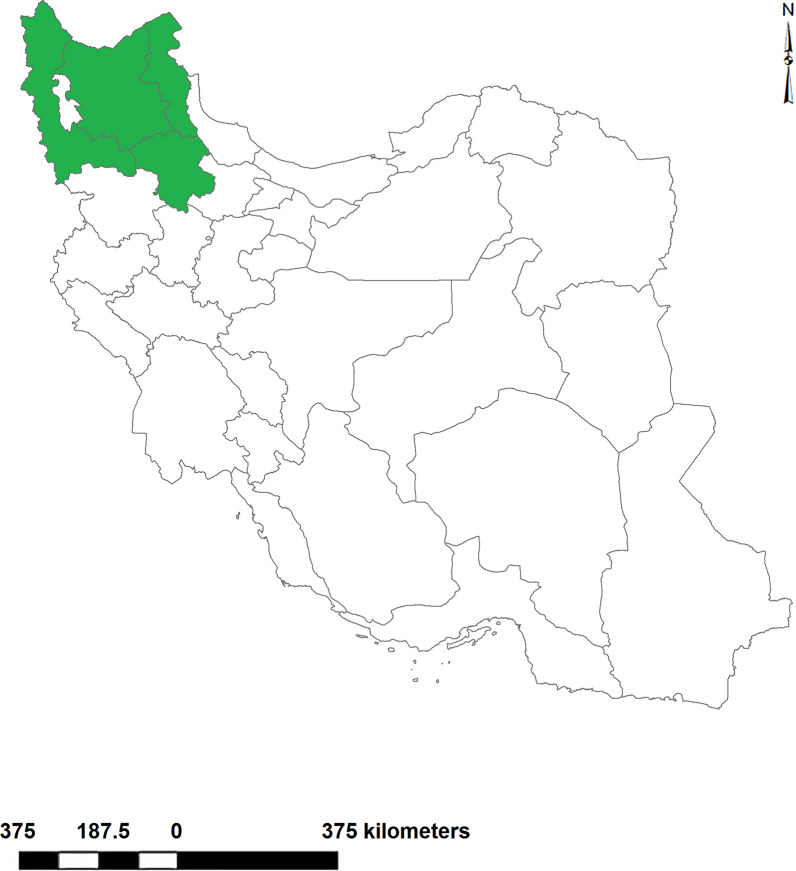
Geographical location of the sampling areas for lymnaeid species in Iran. The provinces surveyed for this study are shown in dark green

This study was conducted in the northwestern region of Iran, an area between latitudes 35.5511 and 39.7820 N and longitudes 44.0325 and 49.4337 E, which includes four provinces of West Azerbaijan, East Azerbaijan, Ardabil, and Zanjan (Fig. 1).

The study areas border Turkey and Iraq in the west; Armenia, the Republic of Azerbaijan, and the Nakhchivan Autonomous Republic of Azerbaijan in the north; Gilan Province, the most important center of human fascioliasis in Iran, in the east; and Kurdistan, Qazvin, and Hamedan Provinces in the south. The climate of the study area varies from Mediterranean and temperate climates in the plains to cold and snowy mountainous climate.

### Snail collection and identification

Based on the epidemiological pattern of fascioliasis in Iran [11, 12] and the distribution of lymnaeid snails in habitats [13, 14], locations near the final hosts (livestock) such as rivers, swamps, bogs, riverbanks, ponds, creeks, streams, and marshes were selected for sampling. Freshwater lymnaeid snails were collected by searching 33 permanent and seasonal habitats in the northwestern provinces of Iran from June to November 2021. A total of 2000 lymnaeid snails were collected manually using a sweeping net (Table 1). The collected samples were placed in bottles containing snail habitat water and were transferred to the parasitology laboratory at the Medical School of Zanjan University of Medical Sciences, where they were identified morphologically according to the keys provided by Pfleger [15]. Since it is not possible to differentiate some lymnaeid species such as *G. truncatula* and* G. schirazensis* by morphological methods [16], it was necessary to use the DNA sequencing method to differentiate these species. In this study, DNA sequencing of all snails was not possible, so the information about these two species is shown as *G. truncatula/G. schirazensis*.

**Table 1:** Geographical coordinates of collected snails and information on isolated *Fasciola* cercariae

Site no.	Latitude; longitude	Altitude (m)	Habitat	Lymnaeids (no.)	Isolated cercariae
1	39.1992° N; 45.1479° E	780	River	*G. t/G. sch* (30)	–
2	38.8918° N; 45.6113° E	827	Riversides	*R. euphratica* (50)	*F. hepatica*
3	38.4605° N; 45.5334° E	1126	River	*R. euphratica* (50)	*F. gigantica*
4	38.4338° N; 44.9127° E	1180	River	*R. euphratica* (30)	–
5	38.5249° N; 44.9215° E	1180	River	*R. euphratica* (18) *G. t/G. sch* (32)	*F. hepatica* *F. hepatica*
6	38.4333° N; 47.6636° E	1250	Bog	*R. euphratica* (20) *G. t/G. sch* (80)	– *F. hepatica*
7	38.5379° N; 48.1395° E	1256	River	*G. t/G. sch* (75)	–
8	38.3788° N; 48.2500° E	1290	Bog	*R. euphratica* (100)	*F. hepatica*
9	37.4230° N; 45.1482° E	1305	Swamp	*R. euphratica* (35) *L. stagnalis* (65)	– *F. hepatica*
10	38.6891° N; 44.9274° E	1308	Pond	*R. euphratica* (50)	*F. hepatica*
11	37.4681° N; 45.1366° E	1310	Pool/bog	*R. euphratica* (40) *L. stagnalis* (35)	*F. gigantica* –
12	38.1143° N; 46.2345° E	1339	Bog	*R. euphratica* (50)	*F. hepatica*
13	38.3769° N; 48.5442° E	1352	Pool/bog	*R. euphratica* (50)	*–*
14	38.1990° N; 48.2335° E	1384	River	*R. euphratica* (75)	*F. hepatica*
15	38.4756° N; 48.2469° E	1448	Swamp	*R. euphratica* (40)	*F. hepatica*
16	38.2592° N; 44.7733° E	1450	Swamp	*R. euphratica* (12) *G. t/G. sch* (58)	*F. gigantica* *–*
17	38.0957° N; 48.1141° E	1476	River	*G. t/G. sch* (60)	*–*
18	38.3510° N; 45.2731° E	1502	Bog	*R. euphratica* (20) *G. t/G. sch* (80)	*F. hepatica* *F. hepatica*
19	36.7455° N; 48.2503° E	1506	Bog	*G. t/G. sch* (50)	*F. hepatica*
20	37.7205° N; 44.7232° E	1511	River	*R. euphratica* (15)	*–*
21	36.7596° N; 48.3782° E	1590	Pool/bog	*R. euphratica* (75)	*F. hepatica*
22	36.6661° N; 48.4497° E	1593	Stream	*R. euphratica* (5)	*–*
23	36.6638° N; 48.5018° E	1645	River	*R. euphratica* (50)	*–*
24	37.4872° N; 47.0215° E	1658	Creek	*R. euphratica* (50)	*F. gigantica*
25	36.6195° N; 48.5367° E	1708	Ditch	*R. euphratica* (50)	*F. hepatica*
26	37.9373° N; 47.6599° E	1710	River	*R. euphratica* (100)	*F. hepatica*
27	38.8187° N; 44.5495° E	1740	River	*G. t/G. sch* (40)	*F. hepatica*
28	36.5123° N; 48.7766° E	1765	River	*G. t/G. sch* (50)	*F. hepatica*
29	36.4480° N; 48.7917° E	1776	Stream	*R. euphratica* (75)	*–*
30	36.7892° N; 48.5617° E	1950	Reservoir	*R. euphratica* (95)	*F. gigantica*
31	36.7137° N; 48.5635° E	1996	Pond	*R. euphratica* (50)	*–*
32	37.7327° N; 46.1494° E	2012	Pond	*R. euphratica* (25) *G. t/G. sch* (75)	*F. gigantica* –
33	38.1437° N; 44.4510° E	2319	Reservoir	*R. euphratica* (40)	*F. hepatica*

Lymnaeids (no.): In this column, the frequency of different lymnaeid species collected at each collection point is shown

*G. t/G. sch*: *G. truncatula/G. schirazensis*; *R. euphratica*: *Radix euphratica*; *L. stagnalis*: *Lymnaea stagnalis*; *F. hepatica*: *Fasciola hepatica*; *F. gigantica*: *Fasciola gigantica*

### Examination of cercariae from snails

After 2 days of acclimatization to laboratory conditions, the snails were divided into groups of eight inside Petri dishes containing 20 ml filtered water originating from a pond. These dishes were placed at 25–30 °C under artificial light for at least 2 h or overnight to induce shedding. After the incubation, the Petri dishes were examined under a stereomicroscope for the presence of cercariae. Snails from positive batches were tested individually to identify the infected snails. Emerged cercariae were collected in dechlorinated water and observed for their swimming behavior and then photographed and preserved in 96% ethanol at −20 °C for molecular investigations. Several cercariae isolated from each snail were stained with Ehrlich's alum hematoxylin and eosin and were classified using a light microscope according to Frandsen and Christensen [17].

Also, several collected snails were crushed against a glass plate and then examined with a stereomicroscope for trematode cercariae, rediae, or sporocysts. Snails infected with trematode larval stages were preserved in individual containers using 96% ethanol for molecular investigations.

### DNA extraction and PCR–RFLP of ITS1 rDNA for *Fasciola* species identification

**Fig. 2:**
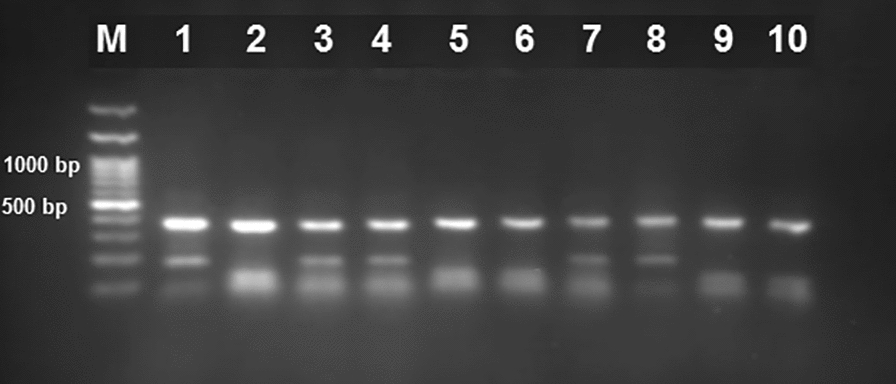
PCR–RFLP pattern of the *ITS1* gene of *Fasciola *spp. in infected snails: *F. gigantica* (lanes 2, 4, 5, 8, 9) and *F. hepatica* (lanes 3, 6, 7, 10, 11), 100-bp DNA size marker (lane 1), and negative control (lane 12)

The ethanol-preserved cercariae were subjected to DNA extraction using a SamBio kit (Ambio^®^, Sambio™, Iran), according to the manufacturer’s instructions. Amplification of the internal transcribed spacer 1 (*ITS1*) fragment with genus-specific primers was used to identify *Fasciola* cercariae isolated from snails. PCR amplification of *ITS1* of the ribosomal DNA (fragment 680 base pairs [bp] in length) was performed using primers ITS1-F (5′-TTGCGCTGATTACGTCCCTG-3′) and ITS1-R (5′-TTGGCTGCGCTCTTCATCGAC-3′) [18]. The PCR reaction was carried out in a total volume of 25 μl, containing 10 pmol of each primer, 5 μl template DNA, 10 µl of master mix (GeneDireX, Taiwan) and 8 μl double-distilled water (ddH_2_O). PCR conditions were adjusted by pre-denaturation at 95 °C for 4 min, denaturation at 95 °C for 60 s, annealing at 55 °C for 45 s, elongation at 72 °C for 60 s in 35 cycles, and post-elongation at 72 °C for 10 min. In each PCR run, ddH_2_O was used instead of the DNA template as negative control. The PCR products were separated on agarose gel (1.5%) and visualized using a transilluminator. Restriction fragment length polymorphism (RFLP) analysis was conducted to differentiate *F. hepatica* from *F. gigantica* in the *ITS1* gene [19] using the RsaI restriction enzyme (Thermo, Germany). A total volume of RFLP reaction contained 1 μl RsaI enzyme, 1 μl buffer, 5 µl PCR product, and nuclease-free water for a final volume of 25 μl. The mixture was incubated at 37 °C for 16 h. The products were run on 2% agarose gel, stained with safe stain, and visualized by an ultraviolet (UV) transilluminator. PCR–RFLP produced the three expected restriction fragment sizes of 360, 100, and 60 bp for *F. hepatica*, and three fragment sizes of 360, 170, and 60 bp for *F. gigantica* [19] (Fig. 2).

### Geospatial data

The latitude–longitude coordinates and altitude of the collection sites of *Fasciola*-infected and noninfected snails were retrieved from global positioning system (GPS) data (Premium GPS Map Camera version 1.3.6) and imported into an Excel file. The file was exported to ArcMap 10.5 (http://www.esri.com/arcgis) software and the attribute point shape file layer was generated. This layer encompassed all points where snails had been gathered. The weather temperature (mean, minimum, and maximum values), soil temperature, dew temperature, rainfall, humidity (mean, minimum, and maximum values), evaporation, air pressure (mean, minimum, and maximum values), and wind speed data were recovered from 57 weather synoptic stations for 1 year (2021) in the four provinces of Ardebil, Azarbayjan-e-Sharghi, Zanjan, and Azarbayjan-e-Gharbi. Digital elevation model (DEM) raster and land cover vector layers were recovered from the natural resources departments of the four investigated provinces. Then they were merged to generate one layer that covered the entire study area.

### Geoclimatic analysis

After examination of errors of different interpolation methods, the raster layers of isothermal, isohydral, and evaporation were generated by the tension-based spline interpolation method. The different pressures and wind speed layers were made by kriging and humidity layer by inverse distance weighted (IDW) interpolation methods with a resolution grid of 2 × 2 km. The point shape file layer including 33 sampling points was extracted with the geoclimatic and DEM raster layers. The identity tool was used to calculate the geometric intersection of the layer obtained from the extraction of all raster layers with land cover (polygonal) vector layer to develop the final layer in which each point has the properties of all the overlapped identity features from the abovementioned raster and vector layers. The analysis was done using ArcGIS version 10.5. The attribute of this layer was converted to an Excel format and exported to SPSS software for statistical analysis.

### Statistical analysis

Statistical analyses were performed between areas where snails infected with *Fasciola* cercariae were found and those with noninfected snails. They were also realized between areas with snails infected with *F. hepatica* and those with snails infected with *F. gigantica* using the *t*-test and Chi-square test. The analyses were performed using IBM SPSS version 21 software.

## Results

The collected lymnaeid snails were identified as *R. euphratica*, *G. truncatula/G. schirazensis*, and *L. stagnalis*. PCR–RFLP of the *ITS1* fragment of cercariae isolated from 38 lymnaeid snails showed that 19 snails were infected with *F. hepatica* and six snails were infected with *F. gigantica* (Table 1).

The three values of the meteorological temperature, soil temperature, and dew temperature between the points with uninfected snails and those with snails harboring *Fasciola* cercariae showed no significant differences. Similarly, average rainfall and the three values of moisture and evaporation between these geographical points were not different. The same applies to the three values of atmospheric pressure and wind speed. The average elevation between these points was not statistically different (Table 2). Finally, the Chi-square test showed no significant difference between points with uninfected snails and those with individuals harboring *Fasciola* cercariae compared to the land cover where these points are distributed (Table 3).

**Table 2:** Results of geoclimatic factors of *Fasciola*-infected and noninfected points

Variable name		Mean	SD	*P*-value	CI (95%)	
Mean temperature (°C)	Infected points	12.68	1.6	0.75	−1.04	1.42
	Noninfected points	12.87	1.53			
Maximum temperature (°C)	Infected points	18.72	1.79	0.62	−1.43	2.3
	Noninfected points	19.16	2.47			
Minimum temperature (°C)	Infected points	6.21	2.01	0.65	−0.99	1.56
	Noninfected points	6.49	1.44			
Soil temperature (°C)	Infected points	3.22	2.02	0.36	−0.9	2.33
	Noninfected points	3.93	2.02			
Dew temperature (°C)	Infected points	1.47	0.76	0.22	-0.32	1.29
	Noninfected points	1.95	1.07			
Rain (mm)	Infected points	233	95.15	0.77	−102.9	78.21
	Noninfected points	221	118.18			
Mean humidity (%)	Infected points	52.52	5.44	0.54	−2.94	5.44
	Noninfected points	53.77	5.19			
Maximum humidity (%)	Infected points	92.2	3.96	0.18	−1.09	5.41
	Noninfected points	94.35	4.11			
Minimum humidity (%)	Infected points	15.68	1.92	0.82	−2.96	2.4
	Noninfected points	15.4	3.65			
Evaporation (mm)	Infected points	1648.73	336.89	0.42	−320.94	138.84
	Noninfected points	1557.68	270.293			
Mean air pressure (hPa)	Infected points	1012.80	0.45	0.98	0.41	0.4
	Noninfected points	1012.80	0.52			
Maximum air pressure (hPa)	Infected points	861.02	16.52	0.83	−14.26	17.31
	Noninfected points	862.54	20.61			
Minimum air pressure (hPa)	Infected points	860.45	14.17	0.95	−13.67	12.98
	Noninfected points	860.11	17.35			
Wind velocity (km/h)	Infected points	2.91	0.66	0.6	−0.49	0.82
	Noninfected points	3.07	0.86			
Elevation (m)	Infected points	1506.39	325.14	0.68	−318.49	214.48

Mean: The average number

*SD* standard deviation, *CI* confidence interval

**Table 3:** Distribution of uninfected and *Fasciola*-infected points in different types of land cover

	Jungle	Rangeland	Cold and semi-condensed rangeland	Irrigated farm	Dry farm	Sandy land	Good and steppe rangeland	Around large water bodies	Total	*P*-value
Infected points	0	0	4 (17.4%)	7 (30.4%)	7 (30.4%)	1 (4.3%)	3 (13%)	1 (4.3%)	23 (100%)	0.54
Noninfected points	1 (10%)	1 (10%)	1 (10%)	2 (20%)	3 (30%)	1 (10%)	1 (10%)	0	10 (100%)	
total	1	1	5	9	10	2	4	1	33	

Rangeland: Grasslands, shrublands, woodlands, wetlands, and deserts that are grazed by domestic livestock or wild animals

**Fig. 3:**
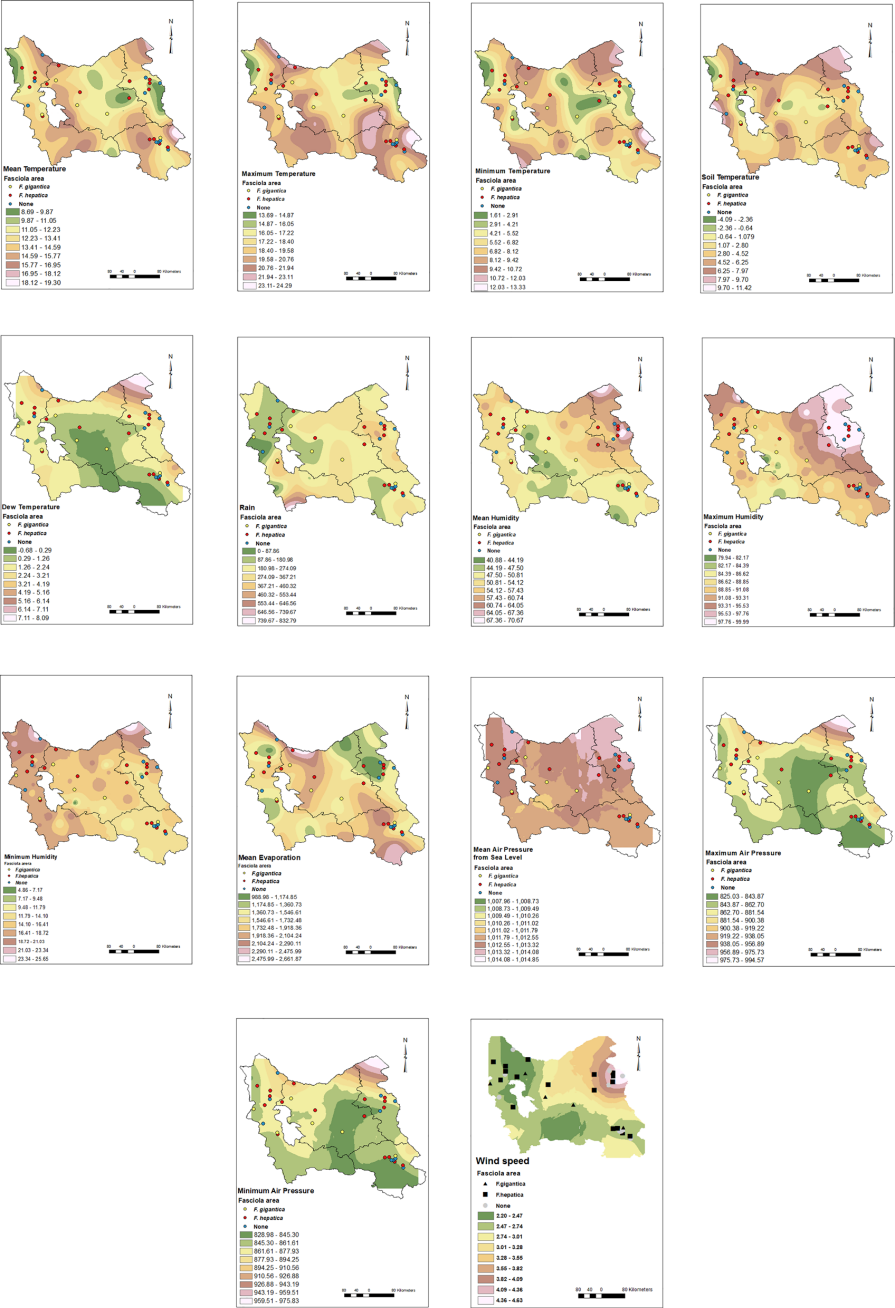
Climate interpolated raster maps: minimum air pressure (**a**), maximum air pressure (**b**), mean air pressure (**c**), minimum temperature (**d**), maximum temperature (**e**), mean temperature (**f**), minimum humidity (**g**), maximum humidity (**h**), mean humidity (**i**), soil temperature (**j**), rain (**k**), dew temperature (**l**)

**Fig. 4:**
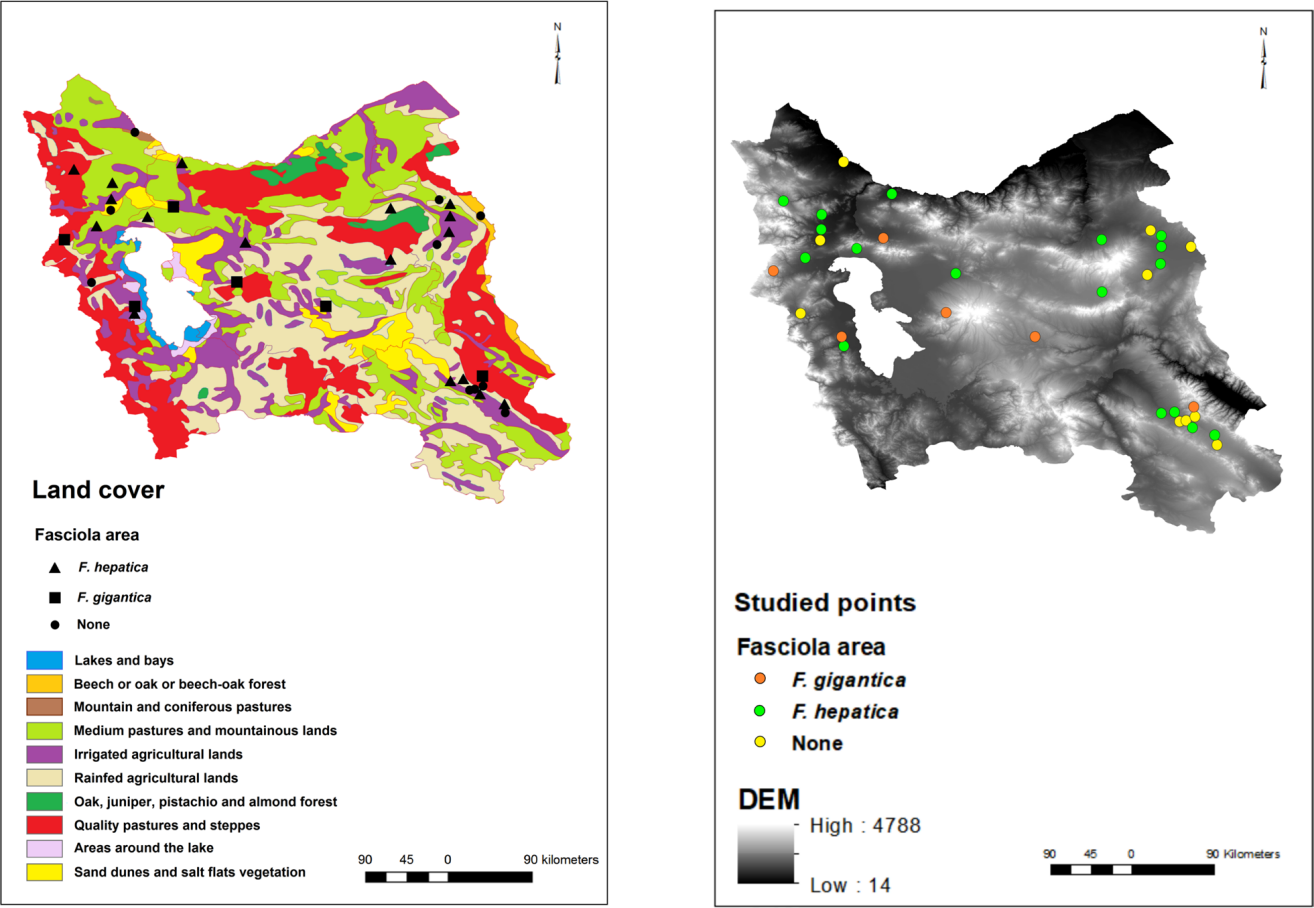
Maps of the environmental factors: digital elevation model (**a**) and land cover (**b**)

Statistical analyses of geoclimatic characteristics of *F. hepatica*- and *F. gigantica*-prevalent points showed that the mean humidity and maximum humidity were both different between those areas (*P* < 0.05), while minimum humidity, rainfall, and evaporation were not associated with the presence of *Fasciola* species. Compared with areas with *F. gigantic*-infected snails, the humidity was higher in *F. hepatica*-infected areas (Fig. 3). Moreover, wind speed showed a significant difference (*P* < 0.05) between *F. hepatica*- and *F. gigantica*-infected points, and it was higher in *F. hepatica*-infected areas. Although elevation was not significantly different between *F. hepatica*- and *F. gigantica*-infected points, a trend was found for this factor, as the areas of *F. hepatica* were about 300 m lower than those of *F. gigantica* (Fig. 4 and Table 4). Finally, no difference was found between *F. hepatica*- and *F. gigantica*-infected areas in terms of land cover (Fig. 4 and Table 5).

**Table 4:** Geoclimatic characteristics of farms where snails infected with *F. hepatica* and *F. gigantica* were found

Variable name		Mean	SD	*P*-value
Mean temperature (°C)	*F. hepatica*	12.69	1.77	0.94
	*F. gigantica*	12.65	1.07	
Maximum temperature (°C)	*F. hepatica*	18.74	2	0.89
	*F. gigantica*	18.65	1.14	
Minimum temperature (°C)	*F. hepatica*	6.18	1.94	0.92
	*F. gigantica*	6.28	2.38	
Soil temperature (°C)	*F. hepatica*	3.24	2.07	0.92
	*F. gigantica*	3.14	2.05	
Dew temperature (°C)	*F. hepatica*	1.61	0.75	0.84
	*F. gigantica*	1.01	0.65	
Rain (mm)	*F. hepatica*	235.71	92.82	0.86
	*F. gigantica*	227.06	110.45	
Mean humidity (%)	*F. hepatica*	53.45	5.98	0.046*
	*F. gigantica*	49.89	2.08	
Maximum humidity (%)	*F. hepatica*	92.94	4.26	0.042*
	*F. gigantica*	90.08	1.96	
Minimum humidity (%)	*F. hepatica*	15.65	2.01	0.92
	*F. gigantica*	15.74	1.84	
Evaporation (mm)	*F. hepatica*	1632.66	376.98	0.62
	*F. gigantica*	1694.28	203.12	
Mean air pressure (hPa)	*F. hepatica*	1012.84	0.49	0.41
	*F. gigantica*	1012.69	0.35	
Maximum air pressure (hPa)	*F. hepatica*	862.37	17.64	0.47
	*F. gigantica*	857.19	13.46	
Minimum air pressure (hPa)	*F. hepatica*	861.16	15.38	0.65
	*F. gigantica*	858.46	10.94	
Wind velocity (km/h)	*F. hepatica*	3.02	0.74	0.044*
	*F. gigantica*	2.6	0.16	
Elevation (m)	*F. hepatica*	1429.25	239.9	0.053*
	*F. gigantica*	1724.96	451.03	

*SD* standard deviation. *Indicates significance at *P* < 0.05

**Table 5:** Frequency of grazing types on farms infected with *F. hepatica* and *F. gigantica*

	Cold and semi-condensed rangeland	Irrigated farm	Dry farm	Sandy land	Good and steppe rangeland	Around large water bodies	Total	*P*-value
*F. hepatica*	4 (23.5%)	6 (35.3%)	5 (29.4%)	0	1 (5.9%)	1 (5.9%)	17 (100%)	0.17
*F. gigantica*	0	1 (16.7%)	2 (33.3%)	1 (16.7%)	2 (33.3%)	0	6 (100%)	
Total	4	7	7	1	3	1	23	

## Discussion

Considering the role of snails in the transmission of important human parasites such as *Fasciola*, *Schistosoma*, and *Clonorchis*, it is very important to study the population distribution of host snails, the various factors affecting their distribution, and the extent of their contamination [20, 21]. The occurrence cycle of fascioliasis, as one of the most important diseases transmitted by snails, is largely related to snails of the Lymnaeidae family [1]. Several studies have investigated the relationship between climate change and parasitic diseases, especially new and re-emerging diseases [22–24]. According to the research, it seems that in fascioliasis, the geoclimatic and environmental characteristics have several effects on larval forms of *Fasciola* and on lymnaeid snails [25–27]. Also, the relationship between the emergence of fascioliasis and aspects of global changes such as import/export and livestock management, changes in the human environment, travel, and changes in human nutritional habits (diet) has been investigated [1, 28, 29].

Among the environmental and geoclimatic factors, altitude is an important factor in the prevalence of fascioliasis. Different results have been obtained in the studies on the effect of altitude on the spread of fascioliasis and *Fasciola* species. In a study using the geographical information system (GIS) in the south of Brazil, a higher percentage of infection with *F. hepatica* was reported in cattle in low-altitude areas, about 150 m above sea level [30], while the highest prevalence of human fascioliasis was reported in the very high-altitude areas of the northern Bolivian Altiplano at an altitude between 3800 and 4100 m above sea level [31]. In the present study, fascioliasis was observed in lymnaeid snails at altitudes between 827 and 2319 m in the northwestern regions of Iran. Although no significant difference was observed between the elevation of *F. hepatica*- and *F. gigantica*-infected areas, *F. gigantica*-infected areas were about 300 m higher than the *F. hepatica*-infected areas. However, in a study conducted on the relationship between altitude and the prevalence of *F. hepatica* and *F. gigantica* in the Nile River basin in Ethiopia, researchers using GIS modeling concluded that the infection rate of *F. gigantica* in livestock living in the area in question decreased with increasing elevation, while the opposite occurred in the case of *F. hepatica* [32]. In a study by Ashrafi et al. [33] on fascioliasis in ruminants in Gilan province of Iran, the prevalence of *F. gigantica* in areas below sea level was significantly higher than that of *F. hepatica*, while in areas with an altitude of more than 100 m, the prevalence of *F. hepatica* was significantly higher than that of *F. gigantica*. Considering that in this study the intermediate host snails of these two species of *Fasciola* were not investigated, it is difficult to compare its results with the present research. However, it is possible that the difference in the environmental fauna and the average height of the study areas in these two studies influenced this difference. Also, the extreme events related to climate change in the study areas of the present research, which were responsible for the superiority of the distribution of *R. euphratica* as the main intermediate host of *F. gigantica* [34], should be considered.

Contrary to the results of the current study, the prevalence of *F. gigantica* in Ugandan buffaloes living below 1500 m was significantly higher than that in areas above 1500 m [35]. These results could be related to the high prevalence of the parasite in *Ligia natalensis*, the intermediate host of *F. gigantica*, at low altitude in the area studied in Uganda. In comparison, it appears that the higher prevalence of *F. gigantica* in high-altitude Iranian areas is also related to the frequency of the host snail in this study. In fact, it can be argued that the prevalence of the two species of *Fasciola* in snails living at different altitudes is directly related to the ecology of the host snail. Temperature and humidity are also important influencing factors in the spread of fascioliasis. According to a report by Rojo-Vazquez et al. [36], the seasonality of fascioliasis is closely related to rainfall and temperature, and even slight changes in these factors may directly affect the life cycle of the parasite in the intermediate hosts. By modeling the effects of climate change and predicting the risk factors affecting the spread of *F. hepatica* using GIS, Fox et al. [37] concluded that fascioliasis outbreaks in areas with higher and longer annual precipitation, high soil moisture, and excess water will likely be more numerous. In the present study, humidity was found to be a determining factor in the spread of *F. hepatica* and *F. gigantica*, as humidity was significantly higher in areas infected with *F. hepatica* than in the areas where *F. gigantica* was found. In this research, the wind speed in the areas where the snails were infected with *F. hepatica* was significantly higher than the areas where the snails were infected with *F. gigantica*. Due to the lack of information about the effect of wind speed on the prevalence of fascioliasis, there is a need for more research on this topic in fascioliasis-endemic areas. The analysis of other environmental and climatic factors investigated in the present study did not show any significant difference between the areas infected with fascioliasis and those not infected with it, and between the two species of *Fasciola* studied.

According to Bargues et al. [16], the high phenotypic similarity and genotypic differences between *G. schirazensis* and *G. truncatula* is an important issue in the molecular epidemiology of fascioliasis. These researchers consider it necessary that a large body of literature on *G. truncatula* should be revised; although *G. truncatula* is a known host of *F. hepatica*, the role of *G. schirazensis* as a host for *Fasciola *spp. has not been proven [20]. Therefore, the sympatric distribution of these two species of snails can interfere in estimating the epidemiology of fascioliasis [38]. Although in this study the molecular differentiation of these two species of lymnaeid snails was not performed, it seems not to affect the final analysis of bioclimatic factors on the prevalence of fascioliasis in these snails.

## Conclusions

Most of the research conducted on the relationship between climatic and geographical factors with the prevalence of different *Fasciola* species has been done on the final hosts of the parasite. However, considering the direct relationship of many of these factors with the ecology and biological conditions of the snail host, it seems that more research is needed in this field. According to this aim, the present study shows the relationship between a number of climatic and geographical factors including humidity, wind speed, and to some extent altitude with the differences in the prevalence of *F. hepatica* and *F. gigantica* in the northwestern regions of Iran. Further research is recommended on the relationship between environmental and climatic factors and the contamination of intermediate host snails with different *Fasciola* species.

## Data Availability

Data supporting the conclusions of this article are included within the article.

## Abbreviations

GIS: Geographical information systems
PCR: Polymerase chain reaction
RFLP: Restriction fragment length polymorphism
rDNA: Ribosomal DNA
IDW: Inverse distance weighted
DEM: Digital elevation model
ITS1: Internal transcribed spacer 1
GPS: Global positioning system
